# Vitamin D and 1-hour post-load plasma glucose in hypertensive patients

**DOI:** 10.1186/1475-2840-13-48

**Published:** 2014-02-20

**Authors:** Angela Sciacqua, Maria Perticone, Nadia Grillo, Tania Falbo, Giuseppe Bencardino, Elvira Angotti, Franco Arturi, Giuseppe Parlato, Giorgio Sesti, Francesco Perticone

**Affiliations:** 1 Department of Medical and Surgical Sciences, University Magna Græcia of Catanzaro, V.le Europa 88100, Catanzaro, Italy; 2 Clinic Chemical Unit, University Hospital of Catanzaro, Catanzaro, Italy

**Keywords:** Vitamin D, Glucose tolerance, Insulin resistance

## Abstract

**Background:**

A plasma glucose value ≥155 mg/dl for 1-hour post-load plasma glucose during an oral glucose tolerance test (OGTT) is able to identify subjects with normal glucose tolerance (NGT) at high-risk for type-2 diabetes and with subclinical organ damage. We designed this study to address if 25-hydroxyvitamin D [25(OH)D] circulating levels are associated with glucose tolerance status, and in particular with 1-hour post-load plasma glucose levels.

**Methods:**

We enrolled 300 consecutive Caucasian hypertensive never-treated outpatients (160 men and 140 women, aged 52.9 ± 9.2 years). Subjects underwent OGTT and measurements of 25(OH)D and standard laboratory tests. Estimated glomerular filtration rate (e-GFR) was calculated by CKD-EPI formula and insulin sensitivity was assessed by Matsuda-index.

**Results:**

Among participants, 230 were NGT, 44 had impaired glucose tolerance (IGT) and 26 had type-2 diabetes. According to 1-h post-load plasma glucose cut-off point of 155 mg/dL, we divided NGT subjects into: NGT < 155 (n = 156) and NGT > 155 mg/dL (n = 74).

NGT ≥ 155 had higher significant fasting and post-load glucose and insulin, parathyroid hormone and hs-CRP levels than NGT < 155. On the contrary, Matsuda-index, e-GFR, and 25(OH)D were significantly lower in NGT ≥ 155 than NGT < 155 subjects. In the multiple regression analysis, 25(OH)D levels resulted the major determinant of 1-h post-load plasma glucose in all population and in the four groups of glucose tolerance status. In the whole population, Matsuda-index, hs-CRP and e-GFR explained another 12.2%, 6.7% and 1.7% of its variation.

**Conclusions:**

Our data demonstrate a significant and inverse relationship between 25(OH)D levels and glucose tolerance status, particularly with 1-h post-load glucose.

## Background

Vitamin D is an important micronutrient involved in calcium homeostasis, musculoskeletal health and in the pathophysiology of different organ systems. In fact, vitamin D receptors have an extensive tissue distribution including skeletal muscle, vascular smooth cells, cardiomyocytes, endothelium and pancreatic β-cells [1,2]. Recently, some experimental and clinical data showed a strong association between hypovitaminosis D and different clinical conditions, suggesting the hypothesis that vitamin D deficiency plays a crucial role in the pathogenesis of both metabolic and cardiovascular diseases [3-6].

Growing evidences, but not all, indicate that suboptimal vitamin D status plays a role in the development of impaired glucose homeostasis, insulin resistance and type-2 diabetes [7-14] because it causes impairment in insulin receptor expression and insulin secretion in animal models and humans [15,16]. In particular, in a large sample from the Ely study, baseline 25-hydroxyvitamin D [25(OH)D] levels were inversely associated with 10-year risk of hyperglycemia, insulin-resistance and metabolic syndrome [7]. Moreover, higher plasma 25(OH)D concentrations are associated with lower risk of incident type-2 diabetes in women, in a case–control retrospective analysis conducted in a large sample of the Nurses’ Health Study [8]. On the contrary, there are data demonstrating no association between vitamin D levels and glucose tolerance status in the general population [17] and in the obese subjects [18].

Recently, a cut-off point of 155 mg/dL for 1-h post-load plasma glucose value during the oral glucose tolerance test (OGTT) was able to identify subjects with normal glucose tolerance (NGT) but at high risk of type-2 diabetes [19]. In addition, in hypertensive patients, the 1-h post-load plasma glucose value was strongly associated with different subclinical organ damage [20-24] thus increasing risk for future cardiovascular events [25-27].

However, at this moment it is unknown whether vitamin D levels are able to affect 1-h post-load plasma glucose in hypertensive NGT subjects. Thus, we designed this study to evaluate, in a group of newly diagnosed hypertensive subjects, the possible relationship between 25(OH)D levels and post-load plasma glucose.

## Methods

### Study population

To conduct a cross-sectional analysis, we enrolled, during the spring, 300 consecutive hypertensive never-treated outpatients who were free of complications (160 men and 140 women, aged 52.9 ± 9.2 years, age range 36–58 years) and participating in the Catanzaro Metabolic Risk Factors Study (CATAMERIS). All subjects were Caucasian and underwent physical examination and review of their medical history with regard to family history of diabetes. Secondary forms of hypertension were excluded by systematic testing by a standard clinical protocol, including renal U.S. studies, computed tomography, renal scan, catecholamine, plasma renin activity, and aldosterone measurements. Other exclusion criteria were history or clinical evidence of coronary and/or valvular heart disease, congestive heart failure, hyperlipidaemia, peripheral vascular disease, chronic gastrointestinal diseases associated with malabsorption, or chronic pancreatitis; history of malignant disease, alcohol or drug abuse, or liver or kidney failure; and any treatments interfering with glucose or vitamin D metabolism.

All subjects underwent evaluation for weight, height, and body mass index (BMI). After 12-h fasting, a 75-g OGTT was performed with 0, 30, 60, 90, and 120-min sampling for plasma glucose and insulin. Briefly, the test was performed using a glucose load containing the equivalent of 75 g anhydrous glucose dissolved in water. Glucose tolerance status was defined on the basis of OGTT using the World Health Organization criteria. In particular, T2D was defined by a 2-h plasma glucose ≥ 200 mg/dL (11.1 mmol/L), whereas patients with a 2-h plasma glucose between 140 mg/dL (7.8 mmol/L) to 199 mg/dL (11.0 mmol/L) were considered IGT [28]. Insulin sensitivity was evaluated using the Matsuda index (insulin sensitivity index [ISI]), calculated as 10,000/square root of [fasting glucose (mmol/L) × fasting insulin (mU/L)] × [mean glucose × mean insulin during OGTT]. The Matsuda index is strongly related to the euglycemic hyperinsulinemic clamp, which represents the gold standard test for measuring insulin sensitivity [29].

The ethical committee approved the protocol, and informed written consent was obtained from all participants. All the investigations were performed in accordance with the principles of the Declaration of Helsinki.

### Blood pressure measurements

Readings of clinic blood pressure (BP) were measured at 3-minute intervals using a standard sphygmomanometer, and BP values were the average of 3 measurements after a 10-minute period of rest in the supine position. This evaluation was repeated on three separate occasions at least 2 weeks apart. Patients with a clinic systolic BP (SBP) >140 mmHg and/or diastolic BP (DBP) >90 mmHg were defined as hypertensive.

### Laboratory determinations

All laboratory measurements were performed after a fast of at least 12 h. Plasma glucose was determined immediately by the glucose oxidase method (Glucose Analyzer, Beckman Coulter S.p.A.,Milan, Italy). Triglyceride and total, low-density lipoprotein (LDL), and high-density lipoprotein (HDL) cholesterol concentrations were measured by enzymatic methods (Roche Diagnostics GmbH, Mannheim, Germany). Plasma concentrations of insulin and intact parathyroid hormone were determined by chemiluminescence test (Roche Diagnostics). Serum creatinine was measured in the routine laboratory by an automated technique based on the measurement of Jaffe chromogenic assay. C-reactive protein (CRP) was measured by a high-sensitivity turbidimetric immunoassay (Behring, Marburg, Germany). Values of estimated glomerular filtration rate (e-GFR; mL/min/1.73 m^2^) were calculated by using the new equation proposed by investigators in the Chronic Kidney Disease Epidemiology (CKD-EPI) Collaboration [30].

Plasma levels of 25(OH)D were measured by competitive immunoassay with chemiluminescence (CLIA) through the Liaison-Diasorin test, in all patients. Venous samples for the determination of 25(OH)D have been carried out in the morning, on fasting condition. A sample of 2.5 ml of venous blood was collected, and subsequently introduced into disposable test tube equipped with separation accelerator granules. The blood sample was centrifuged for 5 minutes at 2500 rpm in a special centrifuge, 500 μl of the obtained serum were collected and placed in special cuvettes. During the first incubation, the 25(OH)D was dissociated from its binding protein and it was tied to the specific antibody. After 10 minutes, a tracer consisting of isoluminol was added and incubated for another 10 minutes, subsequently the unbound material was removed with a wash cycle. In the last step, the reagents for the chemiluminescence reaction were added. Finally, the signal was measured by a photomultiplier as relative light units (RLU) and it was inversely correlated with the 25(OH)D concentration in each sample. Vitamin D status was classified as follows: deficient (<20 ng/ml), insufficient (20 to <30 ng/ml) and normal (≥30 ng/ml).

### Statistical analysis

Normally distributed data were summarized as mean ± SD, while not normally distributed data as median and interquartile range. Variables having a positively skewed distribution were log transformed (lg10) before the correlational analysis. Considering normally distributed data, ANOVA for clinical and biologic data was performed to test the differences among groups, and the Bonferroni post-hoc test for multiple comparisons was further performed as requested. In addition, Kruskal Wallis test was used to compare the medians among the different groups for not normally distributed data. The χ^2^ test was used for categorical variables. The Pearson method was used to calculate the relationship between 1-h post-load glucose and the following covariates: age, BMI, blood pressure, total and HDL-cholesterol, triglyceride, Matsuda index, high-sensitivity CRP (hs-CRP), e-GFR and 25(OH)D. Variables reaching statistical significance, gender and smoking, as dichotomous values, were inserted in a stepwise multivariate linear regression model to determine the independent predictors of 1-h post-load plasma glucose. The analysis was performed for whole study population and according to different groups of glucose tolerance. Differences were assumed to be significant at P < 0.05. All comparisons were performed using SPSS 20.0 statistical software (SPSS, Inc., Chicago, IL).

## Results

### Study population

Of 300 patients examined by OGTT, 230 (76.7%) were NGT, 44 (14.7%) had impaired glucose tolerance (IGT) and 26 (8.6%) had newly diagnosed type-2 diabetes. A 1-h post-load plasma glucose cut-off of 155 mg/dL during the OGTT was used to stratify NGT subjects into two groups: 156 with 1-h post-load plasma glucose <155 mg/dL (NGT < 155) and 74 with 1-h post-load plasma glucose > 155 mg/dL (NGT ≥ 155). In Table 1 demographic, clinical, and biochemical characteristics of the study groups are reported. There were no significant differences among the groups in gender distribution, age, SBP, DBP, total cholesterol and triglyceride, serum calcium and phosphorus. On the contrary, from the NGT < 155 to the diabetic patients, there was a significant increase in waist circumference, BMI, hs-CRP, creatinine, and a decrease in HDL cholesterol and e-GFR. Obviously, with the worsening of glucose tolerance, there was a progressive increase of fasting, 1-h and 2-h post-load glucose, as well as fasting and 1-h and 2-h insulin values, accounting for the reduction of the Matsuda index/ISI.

**Table 1:** Anthropometric, hemodynamic and biochemical characteristics of the study population according to glucose tolerance status

	**All**	**NGT < 155**	**NGT ≥ 155**	**IGT**	**DM**	P*
**Variables**	**(n = 300)**	**(n = 156)**	**(n = 74)**	**(n = 44)**	**(n = 26)**	
**Gender,***m/f*	160/140	74/82	44/30	26/18	16/10	0.274**
**Age,***years*	52.9 ± 9.2	52.9 ± 8.7	52.7 ± 10.9	53.3 ± 9.4	53.3 ± 7.2	0.984
**Waist,***cm*	91.6 ± 9.1	89.5 ± 7.4	93.5 ± 9.5	94.1 ± 11.6	94.7 ± 9.7	<0.0001
**BMI,***Kg/m*^*2*^	28.1 ± 4.2	27.3 ± 4.0	28.4 ± 4.2	28.8 ± 4.6	29.3 ± 3.9	0.028
**SBP,***mmHg*	141.8 ± 10.7	140.9 ± 8.5	142.4 ± 12.6	143.2 ± 12.8	143.1 ± 12.1	0.463
**DBP,***mmHg*	91.3 ± 6.9	91.4 ± 6.3	91.5 ± 7.3	90.8 ± 7.8	91.4 ± 8.1	0.956
**Fasting glucose,***mg/dl*	94.8 ± 11.5	90.1 ± 7.2	98.6 ± 15.6	99.7 ± 8.3	103.8 ± 11.6	<0.0001
**1-h glucose,***mg/dl*	152.3 ± 48.4	116.1 ± 24.0	182.6 ± 26.2	188.7 ± 39.3	221.6 ± 38.9	<0.0001
**2-h glucose,***mg/dl*	124.7 ± 38.3	105.2 ± 17.6	114.1 ± 18.6	155.6 ± 11.8	220.2 ± 21.7	<0.0001
**Fasting insulin,***μU/ml*	13.5 ± 7.1	11.3 ± 5.4	15.3 ± 7.3	16.3 ± 7.9	16.7 ± 8.9	<0.0001
**1-h insulin,***μU/ml*	111.6 ± 62.1	91.2 ± 54.7	151.8 ± 61.7	115.9 ± 63.1	109.7 ± 50.8	<0.0001
**2-h insulin,***μU/ml*	85.0 ± 56.1	61.8 ± 45.9	97.9 ± 60.3	117.3 ± 57.5	113.1 ± 41.6	<0.0001
**MATSUDA/ISI**	66.5 ± 37.3	83.5 ± 40.8	52.4 ± 25.4	50.5 ± 22.8	44.1 ± 22.5	<0.0001
**Total cholesterol,***mg/dl*	212.5 ± 37.4	211.8 ± 36.7	212.2 ± 40.5	213.6 ± 39.2	215.5 ± 29.6	0.968
**HDL-cholesterol,***mg/dl*	49.6 ± 12.5	51.9 ± 12.3	47.2 ± 10.9	47.7 ± 14.4	45.6 ± 12.1	<0.0001
**Triglyceride,***mg/dl*	122.1 ± 47.6	115.7 ± 40.3	128.4 ± 50.4	129.1 ± 49.8	131.5 ± 69.3	0.106
**hs-CRP,***mg/l*	2.5 ± 1.9	1.8 ± 1.1	3.2 ± 2.1	3.3 ± 2.4	3.7 ± 2.1	<0.0001
**Creatinine,***mg/dl*	0.8 ± 0.2	0.8 ± 0.1	0.9 ± 0.2	0.9 ± 0.2	0.9 ± 0.3	<0.0001
**e-GFR,***ml/min/1.73 m*^*2*^	100.1 ± 25.1	104.1 ± 22.9	96.7 ± 24.6	95.8 ± 29.2	92.4 ± 28.1	0.027
**25(OH)D,***ng/ml*	23.5(18–32.9)	26.5(20–35.6)	21(16–30)	21.5(15.6-28.7)	18.6(13–24.6)	0.002^§^
**Parathyroid hormone,***pg/ml*	62.7 ± 42.7	50.7 ± 37.5	71.6 ± 46.5	77.1 ± 49.9	82.3 ± 19.1	0.004
**Calcium,***mg/dl*	9.4 ± 0.5	9.4 ± 0.3	9.5 ± 0.4	9.3 ± 0.9	9.4 ± 0.4	0.305
**Phosphorus,***mg/dl*	3.3 ± 0.4	3.3 ± 0.4	3.3 ± 0.3	3.3 ± 0.5	3.1 ± 0.4	0.467
**Current smokers,***n(%)*	80(26.7)	45(28.8)	15(20.3)	13(29.5)	7(26.9)	0.750**

*By ANOVA; **By χ^2^ test; § by Kruskal Wallis test.

NGT = normal glucose tolerance; IGT = impaired glucose tolerance; DM = diabetes mellitus; BMI = body mass index; SBP = systolic blood pressure; DBP = diastolic blood pressure; ISI = insulin sensitivity index; hs-CRP = high sensitivity C-reactive protein; e-GFR = estimated glomerular filtration rate.

Moreover, from the NGT < 155 to the diabetic patients there was a progressive decrease of 25(OH)D and an increase of parathyroid hormone levels. Of interest, NGT ≥ 155 subjects had significantly reduced insulin sensitivity and 25(OH)D levels, and an increase of hs-CRP values when compared with NGT < 155; in addition, they had a metabolic and inflammatory profiles similar to IGT and diabetic individuals.

In whole population, 104 (34.7%) patients had a deficient vitamin D status, 104 patients (34.7%) had an insufficient vitamin D status and 92 patients (30.6%) had a normal vitamin D status.

**Figure 1:**
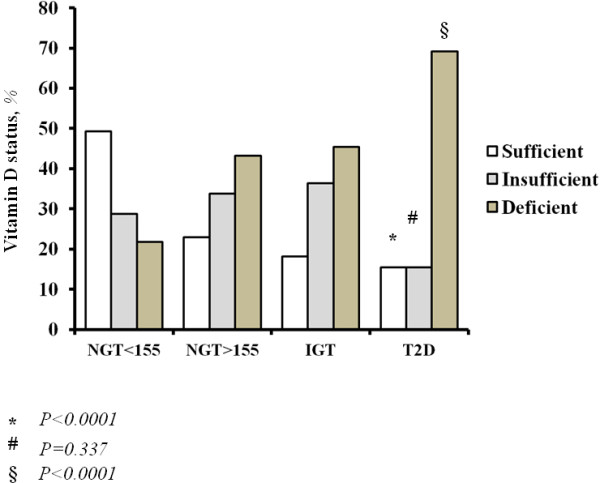
**Vitamin D status according to different groups of glucose tolerance.** In the figure, the vitamin D status, according to different groups of glucose tolerance, is represented. Of interest, vitamin D sufficiency significantly decreases from normal glucose tolerant subjects with 1-hour post-load plasma glucose <155 mg/dl (NGT < 155) to NGT ≥ 155 subjects, impaired glucose tolerant (IGT) subjects and type-2 diabetic (T2D) patients (P < 0.0001). On the contrary the prevalence of vitamin D deficiency significantly increases from the first to the fourth group (P < 0.0001).

Subjects with vitamin D sufficiency significantly decreased from NGT < 155 subjects to diabetic patients (P < 0.0001), while the prevalence of vitamin D deficiency increased from the NGT < 155 to the diabetic patients (P < 0.0001), reaching a 69.2% in the last (Figure 1).

### Correlational analysis

In Table 2 the results of linear correlation analysis, performed to test the correlation between 1-h post-load glucose and different covariates, are reported. In the whole study population, 1-h post-load glucose was inversely correlated with 25(OH)D, Matsuda index, e-GFR and HDL-cholesterol, and it was directly correlated with hs-CRP, BMI and age.

**Table 2:** Correlation analysis between 1-h post load plasma glucose and different covariates in whole study population and in the four groups of different glucose tolerance status

	**All**		**NGT < 155**		**NGT ≥ 155**		**IGT**		**DM**	
	**(n = 300)**		**(n = 156)**		**(n = 74)**		**(n = 44)**		**(n = 26)**	
	***r***	***P***	***r***	***P***	***R***	***P***	***r***	***P***	***r***	***P***
**25(OH)D,***ng/ml*	- 0.439	<0.0001	-0.404	<0.0001	-0.541	<0.0001	-0.480	<0.0001	-0.590	0.004
**Matsuda index/ISI**	-0.418	<0.0001	-0.265	<0.0001	-0.208	0.037	-0.170	0.134	-0.014	0.472
**hsCRP,***mg/dl*	0.384	<0.0001	0.016	0.422	0.240	0.020	0.032	0.418	0.342	0.043
**e-GFR,***ml/min/1.73 m*^*2*^	-0.295	<0.0001	-0.209	0.004	-0.282	0.007	-0.307	0.021	-0.347	0.041
**BMI,***Kg/m*^*2*^	0.188	0.001	0.006	0.471	-0.005	0.484	0.378	0.006	-0.036	0.430
**Age,***years*	0.187	0.001	0.424	<0.0001	0.364	0.001	0.256	0.047	-0.212	0.149
**HDL-cholesterol***mg/dl*	- 0.170	0.002	-0.114	0.078	-0.095	0.209	0.216	-0.080	0.007	0.487
**SBP,***mmHg*	0.097	0.056	0.069	0.197	0.047	0.345	0.048	0.378	0.040	0.423
**Triglyceride,***mg/dl*	0.084	0.073	0.002	0.492	0.113	0.169	0.028	0.427	0.185	0.183
**DBP,***mmHg*	-0.030	0.303	0.010	0.453	-0.072	0.270	0.112	0.235	0.062	0.381
**Total cholesterol***mg/dl*	0.028	0.312	0.018	0.410	-0.020	0.432	0.026	0.433	0.082	0.345

ISI = insulin sensitivity index; hs-CRP = high sensitivity C reactive protein; e-GFR = estimated glomerular filtration rate; BMI = body mass index; SBP = systolic blood pressure; DBP = diastolic blood pressure.

In NGT < 155 subjects, 1-h post-load glucose was directly related with age, and inversely with 25(OH)D, Matsuda index and e-GFR.

The same analysis was performed in NGT ≥ 155 subjects showing that 1-h post-load glucose was inversely correlated with 25(OH)D, Matsuda index and e-GFR, while was linearly correlated with age and hs-CRP.

Similarly, in IGT patients 1-h post-load glucose was inversely correlated with 25(OH)D and e-GFR and it was linearly correlated with BMI, moreover it showed a weak correlation with age.

Finally, diabetic patients showed an inverse correlation between 1-h post-load glucose and 25(OH)D and e-GFR; while it was directly correlated with hs-CRP.

Thus, variables reaching statistical significance and gender, as a dichotomous value, were inserted in a stepwise multivariate linear regression model to determine the independent predictors of 1-h post-load glucose variation (Table 3). In the whole study population, 25(OH)D was the major predictor of 1-h post-load glucose, explaining 19.2% of its variation. Other independent predictors were the Matsuda index, hs-CRP and e-GFR explaining 12.2%, 6.7% and 1.7% of its variation, respectively.

**Table 3:** Predictors of 1-h post load plasma glucose, in whole study population and in groups with different glucose tolerance status

	**All (n = 300)**		**NGT < 155 (n = 156)**		**NGT ≥ 155 (n = 74)**		**IGT (n = 44)**		**DM (n = 26)**	
	***Partial R***^***2***^ ***(%)***	***P***	***Partial R***^***2***^ ***(%)***	***P***	***Partial R***^***2***^ ***(%)***	***P***	***Partial R***^***2***^ ***(%)***	***P***	***Partial R***^***2***^ ***(%)***	***P***
**25(OH)D,***ng/ml*	19.2	<0.0001	12.0	<0.0001	28.1	<0.0001	27.0	0.0001	25.4	0.009
**Matsuda index/ISI**	12.2	<0.0001	18.0	<0.0001	–	–	–	–	–	–
**hs-CRP,***mg/L*	6.7	<0.0001	–	–	–	–	–	–	–	–
**e-GFR,***ml/min/1.73 m*^*2*^	1.7	0.005	–	–	–	–	–	–	–	–
**Age,***years*	–	–	4.0	0.003	5.8	0.015				
**BMI,***Kg/m*^*2*^	–	–	–	–	–	–	8.4	0.026	–	–
***Total R***^***2***^ ***(%)***	39.8	–	34.0	–	33.9	–	35.4	–	25.4	–

hs-CRP = high sensitivity C reactive protein; ISI = insulin sensitivity index; e-GFR = estimated glomerular filtration rate; BMI = body mass index.

In NGT < 155 subjects, the covariates retained in the final model were 25(OH)D, the Matsuda index and age which explained 12%, 18% and 4% of the 1-h post-load plasma glucose variation, respectively. The final model accounted for 34% of its variation.

In NGT ≥ 155 and IGT patients, 25(OH)D was the strongest predictor of 1-h post-load glucose, justifying for 28.1% and 27% of its variation in the respective models. Age in NGT ≥ 155 subjects and BMI in IGT patients added another 5.8% and 8.4% of 1-h post load glucose variation. The final model accounted for 33.9% and 35.4% of 1-h post load glucose variation, respectively.

Finally, in diabetic patients, 25(OH)D was the only independent predictor of 1-h post load glucose justifying 25.4% of 1-h post load plasma glucose variation.

## Discussion

The main novelty obtained in this study is that, in a large population of newly diagnosed hypertensive patients, with different glucose tolerance status, there is an inverse and strong relationship between 25(OH)D levels and 1-h post-load glucose in normo-glycemic subjects. In fact, regarding to post-load plasma glucose, NGT ≥ 155 subjects showed reduced 25(OH)D levels compared to NGT < 155 and similar to those of IGT group. Of interest, this result persists after adjustment for all significant covariates, able to interfere with glycemic metabolism. To our knowledge, this is the first study demonstrating this association that has a clinical importance because NGT subjects are considered at low cardiovascular risk. On the contrary, we have recently demonstrated that NGT with 1-h post-load glucose ≥155 have a worse metabolic and cardiovascular profile, showing a multiple subclinical organ damage [20-24]. Thus, our data consent to reconsider the concept that NGT subjects are a homogeneous group with a low cardiovascular and metabolic risk profile.

Present results may be explained by the pathophysiological mechanisms related to pleiotropic action of vitamin D. In particular, vitamin D may affect both directly, via activation of vitamin D receptors, and indirectly, via regulation of calcium availability, glucose and insulin homeostasis by modulating β-cell function and immune response, and improving peripheral insulin sensitivity [1,2,15]. In keeping with this, there are experimental data demonstrating that hypovitaminosis D causes impairment in insulin receptor expression and insulin secretion in animal models and humans [15,16], while treatment with vitamin D improves β-cell function and glucose tolerance [31,32]. In addition to these actions on glucose metabolism, low levels of vitamin D are associated to the apperance and progression of pro-inflammatory status that, in turn, induces a worsening of glucose tolerance status until the clinical onset of diabetes [33,34]. According with this, NGT ≥155 subjects, both in basal condition and after 1-hour during OGTT, are more insulin-resistant and have higher levels of hs-CRP than NGT < 155. On the basis of these evidences, present data contribute to extend previous knowledge about the association between hypovitaminosis D and cardiovascular events [3-6]. Additional mechanism involved in this association is the regulatory effect of vitamin D on the renin-angiotensin system activity. In fact, there are previously published human and animal studies demonstrating that low plasma vitamin D levels result in up-regulation of the renin-angiotensin system [16,35] that interferes with insulin signaling in different tissues [36-38].

Even if data from several studies have demonstrated that circulating levels of vitamin D are involved in the pathogenesis of type-2 diabetes in humans [7-15], it is probable that other associated functional modifications or clinical conditions contribute to its biological effects. So as observed in our study, vitamin D deficiency is also associated with elevated PTH levels that reproduce a secondary hyperparathyroidism that may contribute, through the reduction of insulin sensitivity and by promoting cardiac and vascular remodeling, to the development of glucose intolerance and cardiovascular complications [39]. Of interest, present data were obtained in subjects with normal renal function. In addition, reduced levels of vitamin D may be operating in diabetes-related obesity; in fact, hypovitaminosis D of obese subjects is due, not only to less exposure to sunlight for the reduction of physical activity, but also because vitamin D is trapped in adipose tissue and it is no longer bioavailable [40,41]. It follows that the biological action of vitamin D is multifactorial and complex, especially as regards to the glucose metabolism. In keeping with this, recently some study have been published with the aim to evaluate the effects of vitamin D supplementation on glucose metabolism and insulin resistance in different setting of patients [42-46]. However, despite the significant increase of vitamin D levels, the absence of consistent results may be explained with the short duration of treatment.

Another important finding emerged from this study is that the number of subjects with sufficient vitamin D levels is low and significantly decreases with the worsening of tolerance status, while the condition of vitamin D deficiency significantly increases. This is a surprise not easily explained, since all our patients live in a sunny area of South Italy and spend many hours outdoors, maybe the season period of study may affect the levels. On the other hand, average levels of vitamin D detected in our subjects are similar to the national average; probably, it is necessary to accurately investigate the causes of this phenomenon.

## Conclusions

Present study might suggest new pathogenetic mechanisms involved in glucose metabolism in particular on post-load glucose, and might contribute to early stratify the cardiovascular and metabolic risk profile at least in hypertensive patients. In particular, our data demonstrate, for the first time, that vitamin D levels are significantly associated with post-load glucose in hypertensive normo-glycemic subjects. Thus, according to previous published evidences, it can be hypothesized that vitamin D deficiency should not be considered only as a feature of osteo-mineral disorders, but also a risk factor for metabolic and cardiovascular diseases. However, if vitamin D supplementation may have a beneficial role on insulin-resistance and glucose homeostasis is currently controversial as shown by a recent meta-analysis of randomized controlled trials, that reported no significant improvement of fasting glucose, glycated haemoglobin or insulin resistance in patients treated with 25(OH)D compared with placebo [47]. Thus, it should be necessary to perform a larger randomized prospective study to clarify this point.

### Limitations of the study

This study has several limitations. At first, this is a cross-sectional study, thus cause-effect relationships cannot be established; moreover the study was conducted in a specific cohort of untreated hypertensive patients, which may be not representative of general population. Finally, the study doesn’t take into account some covariates influencing 25(OH)D levels, so as physical activity and social class.

## Competing interests

All the authors declare that they have no competing interests.

## Authors’ contributions

AS and MP have substantially contributed to conception and design of the study, in the acquisition, analysis and interpretation of data and in drafting the manuscript. NG, TF, GB, EA and FA have contributed to the acquisition, analysis and interpretation of data. GP, GS and FP: have contributed to the interpretation of data, they have been involved in drafting the manuscript and revising it critically. All authors read and approved the final manuscript.
